# Therapeutic Inertia in Dyslipidemia Management for Secondary Cardiovascular Prevention: Results from the Italian ITACARE-P Network

**DOI:** 10.3390/jcm14020493

**Published:** 2025-01-14

**Authors:** Andrea Faggiano, Anna Gualeni, Lucia Barbieri, Gian Francesco Mureddu, Elio Venturini, Francesco Giallauria, Marco Ambrosetti, Matteo Ruzzolini, Francesco Maranta, Maria Vittoria Silverii, Laura Garau, Davide Garamella, Raffaele Napoli, Luigi Maresca, Gaetano Luca Panetta, Antonio Maggi, Stefano Carugo, Francesco Fattirolli, Pompilio Faggiano

**Affiliations:** 1 Department of Cardio-Thoracic-Vascular Diseases, Foundation IRCCS Ca’ Granda Ospedale Maggiore Policlinico, 20122 Milan, Italy; andreafaggiano95@gmail.com (A.F.); lucia.barbieri@policlinico.mi.it (L.B.); laura.garau@unimi.it (L.G.); stefano.carugo@unimi.it (S.C.); 2 Department of Clinical Sciences and Community Health, University of Milan, 20122 Milan, Italy; 3 Cardiovascular Department, Fondazione Poliambulanza, 25124 Brescia, Italy; anna.gualeni@poliambulanza.it (A.G.); antonio.maggi@poliambulanza.it (A.M.); 4 Cardiology and Cardiac Rehabilitation Unit, San Giovanni Hospital Complex, 00184 Rome, Italy; mureddu.gianfra@gmail.com; 5 Unit of Cardiac Rehabilitation, Civil Hospital Cecina (LI), 57023 Cecina, Italy; elio.venturini@uslnordovest.toscana.it (E.V.); davide.garamella@gmail.com (D.G.); 6 Precision Internal Medicine Unit, Department of Translational Medical Sciences, Federico II University of Naples, 80131 Naples, Italy; francesco.giallauria@unina.it (F.G.); raffaele.napoli@unina.it (R.N.); 7 Cardiovascular Rehabilitation Unit, ASST Crema, Rivolta D’Adda Hospital, 26027 Rivolta d’Adda, Italy; marco.ambrosetti@asst-crema.it (M.A.); luigi.maresca@asst-crema.it (L.M.); 8 Cardiology Department, Isola Tiberina-Gemelli Isola Hospital, 00186 Rome, Italy; matteo.ruzzolini@gmail.com (M.R.); gaelu@libero.it (G.L.P.); 9 Cardiovascular Rehabilitation Unit, San Raffaele Scientific Institute, 20132 Milan, Italy; maranta.francesco@hsr.it; 10 SOD Cardiologic Rehabilitation Unit, Careggi University Hospital, 50134 Florence, Italy; mvsilverii@gmail.com (M.V.S.); francesco.fattirolli@unifi.it (F.F.); 11 Department of Experimental and Clinical Medicine, University of Florence, 50134 Florence, Italy

**Keywords:** therapeutic inertia, dyslipidemia, low-density lipoprotein cholesterol, secondary cardiovascular prevention, lipid-lowering therapies

## Abstract

**Background/Objectives:** This study assessed the proportion of secondary cardiovascular prevention patients who achieved low-density lipoprotein (LDL) cholesterol targets as per the 2019 ESC/EAS Dyslipidemia Guidelines. We also evaluated whether lipid-lowering therapies (LLTs) were adjusted in patients not meeting targets and analyzed the likelihood of these modifications achieving recommended levels. **Methods:** A multicenter, cross-sectional observational study retrospectively reviewed medical records of 1909 outpatients in 9 Italian cardiac rehabilitation/secondary prevention clinics from January 2023 to June 2024. Inclusion criteria included prior atherosclerotic cardiovascular disease (ASCVD) and recent LDL-cholesterol levels. Data included demographics, ASCVD presentation, lipid profiles, and LLTs. Patients at very high risk had LDL targets of ≤55 mg/dL, or ≤40 mg/dL for recurrent events within 2 years. Clinicians’ approaches to LLT modification in patients not at target were recorded, with LLT efficacy estimated based on percentage distance from LDL-cholesterol targets. **Results:** Of the 1909 patients, 41.3% met the LDL-cholesterol target. Predictors of achieving targets included male gender, cardiac rehabilitation, recent acute coronary syndrome, diabetes, and triple therapy (statin + ezetimibe + PCSK9 inhibitors). Conversely, a target of ≤40 mg/dL, lack of therapy, and monotherapy were negative predictors. Among 1074 patients not at target, LLT modifications were proposed for 48.6%. Predictors of LLT modification included recent ASCVD events, cardiac rehabilitation, and greater percentage distance from the LDL target, while advanced age and an LDL target of ≤40 mg/dL were negative predictors. However, only 42.3% of modified therapies were predicted to be effective in reaching LDL targets. **Conclusions:** Despite 2019 ESC/EAS guidelines, a significant proportion of high-risk patients did not achieve LDL targets, and proposed LLT modifications were often insufficient. More intensive LLT regimens are needed to improve outcomes in this population.

## 1. Introduction

The 2019 European Atherosclerosis Society/European Society of Cardiology (ESC/EAS) guidelines on dyslipidemias set ambitious low-density lipoprotein (LDL) cholesterol targets for patients in secondary cardiovascular prevention [1]. Specifically, these guidelines recommend that patients at very high cardiovascular risk, such as those with a history of atherosclerotic cardiovascular disease (ASCVD), should maintain LDL-cholesterol levels below 55 mg/dL; for patients with multiple ASCVD events within 2 years, an even lower target of ≤40 mg/dL is suggested [1]. Achieving these stringent targets is crucial in reducing the risk of recurrent cardiovascular events, but real-world evidence, such as the DaVinci Registry [2,3] and Santorini Registry [4], suggests that a substantial proportion of patients fail to reach these levels.

Despite the availability of potent lipid-lowering therapies (LLTs), such as high-intensity statins, ezetimibe, proprotein convertase subtilisin/kexin type 9 serine protease inhibitors (PCSK9i), and newer agents like bempedoic acid and inclisiran, implementation in clinical practice and use of s intensification and combination remain suboptimal [4,5]. Several barriers hinder the effective application of these treatments. One of the most prominent obstacles is therapeutic inertia, defined as the failure to initiate or intensify therapy in a timely manner when indicated [6]. This inertia may stem from both patient and clinician factors, including concerns about drug side effects, lack of awareness of international guidelines recommendations and new therapeutic options, and perceived complexity of treatment regimens [7].

As components of therapeutic inertia, there are structural barriers such as limited access to advanced lipid-lowering agents in certain healthcare settings, high costs, and restrictive reimbursement policies, particularly for newer drugs [8]. Furthermore, adherence to prescribed LLTs is often suboptimal, with patients discontinuing treatment or failing to take medications as directed [9].

Given these challenges, understanding the current level of LDL target achievement in real-world settings and the adequacy of therapy adjustments in patients not at target is critical. This study aimed to evaluate the proportion of patients in a secondary cardiovascular prevention setting who achieved LDL-cholesterol targets as recommended by the 2019 ESC/EAS guidelines. Additionally, we examined whether LLTs were adjusted in patients not meeting these targets and assessed the likelihood that these adjustments were likely adequate and sufficient to achieve the recommended LDL levels.

## 2. Materials and Methods

We conducted a multicenter, cross-sectional observational study. We retrospectively analyzed the medical records of ambulatory outpatients evaluated in nine Italian cardiac rehabilitation programs/secondary cardiovascular prevention clinics from 1 January 2023 to 30 June 2024. At the time of clinical evaluation, the 2019 ESC/EAS guidelines on dyslipidemias, published over 3 years before, had likely been fully implemented in clinical practice. The study was approved by the Ethics Committee of the coordinating center, Fondazione IRCCS Ca’ Granda Ospedale Maggiore Policlinico, Milan, under the protocol named “PolicardioRegistry2022” (#166244). Informed consent was obtained from all participants. All procedures were performed in accordance with the Declaration of Helsinki.

Inclusion criteria were: (1) a prior history of ASCVD; (2) availability of recent serum level of LDL-cholesterol at the time of clinical evaluation; (3) the LDL-cholesterol evaluation occurred at least 4 weeks after both the first ASCVD event and the initiation of LLT. No exclusion criteria based on age or other specific factors were applied in this study.

Data collected included demographics, the presence of traditional cardiovascular risk factors (such as hypertension, dyslipidemia, diabetes, smoking, obesity, chronic kidney disease), and the type of ASCVD presentation (e.g., acute coronary syndrome, atherothrombotic stroke, symptomatic peripheral artery disease, coronary artery bypass graft, percutaneous coronary or peripheral artery intervention, chronic coronary syndrome). Significant carotid and peripheral artery diseases were defined according to the 2017 ESC guidelines [10], as the updated 2024 version was not yet available at the time of the study [11]. We also recorded the time interval between the initial ASCVD presentation and the current clinical evaluation. Patients who experienced at least two ASCVD events within 2 years were identified in line with the 2019 ESC/EAS guidelines [1], which recommend an LDL-cholesterol target of ≤40 mg/dL for such cases. The comprehensive lipid profile, particularly the serum LDL-cholesterol level at the time of clinical evaluation, was recorded along with the current LLT. According to the 2019 ESC/EAS Guidelines [1], all patients included in this study were considered at very high cardiovascular risk due to their secondary cardiovascular prevention status. Consequently, they were considered to have reached their target if their serum LDL-cholesterol was 55 mg/dL or less; for those with recurrent events within a 2-year period, the LDL-cholesterol target was ≤40 mg/dL [1]. For patients not on target for LDL cholesterol, the subsequent clinician’s approach to LLT was recorded. Two groups of patients not at target were identified: those in whom no changes to LLTs were made and those in whom adjustments were implemented, including modifications to the dosage of existing drugs and/or the addition of new lipid-lowering agents, as supported by the literature [12]. To assess the presumed efficacy of changes in LLTs to achieve the LDL-cholesterol target according to 2019 ESC/EAS guideline, we used standard rules based on the evidence from literature: a 6% reduction in LDL-cholesterol by doubling the statin dose, a 20% reduction by adding ezetimibe, a 25% reduction by adding bempedoic acid, and a 50% reduction by adding PCSK9i or inclisiran [13,14,15,16]. Validated correction factors were applied to reported LDL-cholesterol data to estimate the individual basal value, i.e., the estimated LDL-cholesterol in the absence of LLT [17,18].

Furthermore, the distance from the LDL-cholesterol target was calculated and expressed as an absolute value (LDL reported − LDL target, in mg/dL) and as a percentage ((LDL reported − LDL target)/LDL reported × 100). By combining the percentage distance from the target and the expected percentage reduction of LDL-cholesterol achieved after the LLT changes for each not-at-target patient, we estimated whether the proposed change was appropriate and effective. For example, in a patient with an LDL-cholesterol of 67 mg/dL on rosuvastin 10 mg/day (LDL-target: 55 mg/dL, distance from target: 17%), doubling the rosuvastatin dosage was expected to produce only a 6% reduction in LDL-cholesterol (likely ineffective choice), compared to an expected reduction of 20% by adding ezetimibe or bempedoic acid to the existing therapy (likely effective choice).

Using this approach, we calculated how many patients initially not-at-target could have LLT changes prescribed by cardiologists that would be likely to achieve the LDL-cholesterol goal according to ESC/EAS Guidelines.

### Statistical Analysis Methods

The normality of continuous variables was assessed using both the Kolmogorov–Smirnov and Shapiro–Wilk tests. Continuous variables were expressed as mean ± standard deviation (SD) if normally distributed or as median with interquartile range (IQR) if not normally distributed. Categorical variables were presented as frequencies and percentages. For group comparisons, normally distributed continuous variables were analyzed using the Student’s t-test, while non-normally distributed continuous variables were analyzed using the Mann–Whitney U test. Categorical variables were compared using the chi-square test or Fisher’s exact test when appropriate. To identify independent predictors of achieving LDL-cholesterol targets and predictors of therapeutic modifications among patients who did not reach their target, multivariate regression models were used. The models included covariates that were clinically relevant or showed significance in univariate analyses. Results from the regression models were presented as regression coefficients (β). Multicollinearity among variables included in the regression models was assessed using the variance inflation factor (VIF). A VIF > 10 was considered indicative of significant multicollinearity, and such variables were excluded from the final model. For the multivariate regression analysis, the model’s goodness-of-fit was assessed using the F-test to evaluate the overall significance of the model. Additionally, the Hosmer-Lemeshow test was employed to assess the adequacy of the logistic regression model in fitting the data. All statistical analyses were performed using Jamovi 2.3.14. A two-tailed *p*-value of <0.05 was considered statistically significant for all tests.

## 3. Results

The study population included 1909 patients, with medical records collected over an 18-month period from 1 January 2023 to 30 June 2024. Demographic and clinical characteristics of the study population are summarized in Table 1.

The mean age was 68 ± 11.4 years old, and one-fifth of the population was female. Coronary artery disease (CAD) affected 96.5% of the patients, with more than 55% having multivessel CAD. The remaining patients had significant isolated carotid or peripheral artery disease, and approximately 15% of the overall population had vascular disease in 2 or more body districts. The time interval from the initial ASCVD event to the clinical evaluation ranged from 1 to 490 months (mean 77 ± 87 months), with 26.6% of patients being evaluated within one year of their first ASCVD presentation. Multiple ASCVD events within 2 years were reported in 170 patients (8.9%), for whom an LDL-cholesterol target of ≤40 mg/dL is recommended. Lipid profile and LLT data for the entire population are summarized in Table 2. A minority of patients (3.1%) was not receiving any LLT, while only a few were on monotherapy with ezetimibe (1.3%), fibrates (1%), PCSK9i (1.3%) or inclisiran (0.1%). Approximately one-quarter of the population was on statin monotherapy, with 50% on low-to-moderate intensity statins and 50% on high-intensity statins. The remaining patients, accounting for more than 60%, were on combination therapy. The most common regimen was statin plus ezetimibe (54.8%), mainly in a single pill combination, followed by statin plus ezetimibe plus PCSK9i (4.9%), and PCSK9i plus ezetimibe without statin (2%).

**Figure 1:**
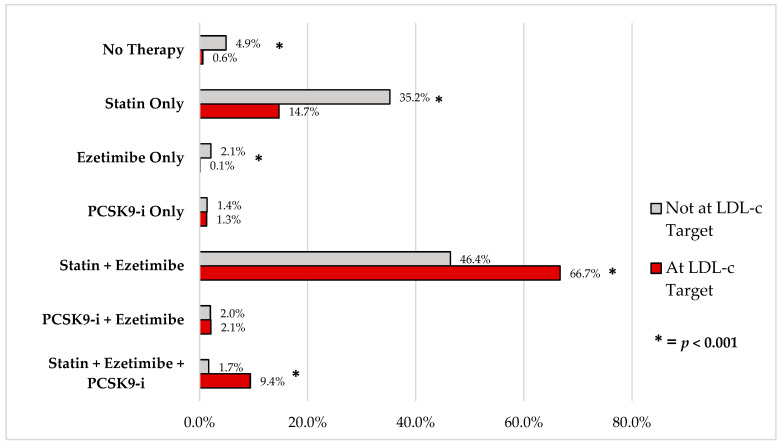
Different lipid-lowering therapies in patients who achieved versus those who did not achieve low-density lipoprotein cholesterol (LDL-c) targets. PCSK9-i: proprotein convertase subtilisin/kexin type 9 serine protease inhibitors.

At the time of evaluation, the LDL-cholesterol target was achieved in 789 out of 1909 patients (41.3%). Specifically, 43.6% of patients with an LDL-cholesterol target of ≤55 mg/dL reached their goal, while only 18.2% of those with a target of ≤40 mg/dL did so. Interestingly, patients evaluated clinically in the first half of 2024 achieved the LDL-cholesterol target more frequently compared to those evaluated in 2023 (44.6% vs. 39.4%, *p* < 0.03). Differences between patients who achieved the LDL-cholesterol target and those who did not are shown in Table 3. The impact of different LLTs on achieving LDL-cholesterol targets is illustrated in Figure 1, showing that combination therapy (statin, ezetimibe, PCSK9 inhibitors) was significantly more effective in reaching LDL-cholesterol targets compared to monotherapy. Table 4 presents the results of the univariate and multivariate analyses for identifying predictors of achieving target LDL-cholesterol levels. The multivariate analysis revealed that male gender, participation in cardiac rehabilitation programs, presentation with recent acute coronary syndrome (ACS), diabetes mellitus, and triple therapy with statins + ezetimibe + PCSK9i were the most consistent predictors of reaching the LDL-cholesterol target. Conversely, a target level of ≤40 mg/dL, absence of therapy, and monotherapy with either statins or ezetimibe were identified as negative predictors for achieving the LDL-cholesterol target.

Among the 1120 patients not meeting the LDL-cholesterol target, information on subsequent changes in therapy was available for 1074 patients. Of these, 522 (48.6%) had changes proposed to their LLT, while 552 (51.4%) did not have any changes proposed. Differences between these two groups are detailed in Table 5. Patients with no proposed LLT changes were older, had a greater time since the initial ASCVD event, and were more likely to have experienced multiple events within 2 years (necessitating an LDL-cholesterol target of ≤40 mg/dL). Additionally, these patients were less frequently enrolled in cardiac rehabilitation programs. Conversely, patients for whom LLT changes have been proposed were more often evaluated within the first year following the initial ASCVD event, had a larger distance from their LDL-cholesterol target, and were more likely to be on the LLT combination.

**Figure 2:**
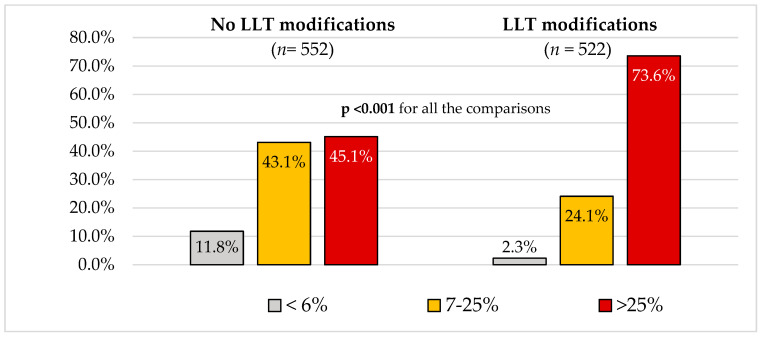
Patient groups stratified by percentage distance from low-density lipoprotein cholesterol (LDL-c ≤ 6%, 7–25%, >25%) and lipid-lowering therapy (LLT) modification status.

In Figure 2, patient groups are categorized based on their percentage distance from the LDL-cholesterol target (≤6%, 7–25%, >25%), with distinctions made between those with and without proposed LLT modification. A larger distance from the target (>25%) was significantly more common among patients with proposed therapy changes, whereas smaller distances from the target (≤6% and 7–25%) were more frequently observed in patients for whom no drug modifications were proposed.

Table 6 showed univariate and multivariate analyses for identifying predictors of LLT modification. The multivariate analysis identified recent ASCVD events (occurring within the past year), enrolment in cardiac rehabilitation programs, and a greater percentage distance from the LDL target as the most consistent predictors for LLT modification. Conversely, advanced age and an LDL target of ≤40 mg/dL were confirmed as negative predictors for LLT modification.

**Figure 3:**
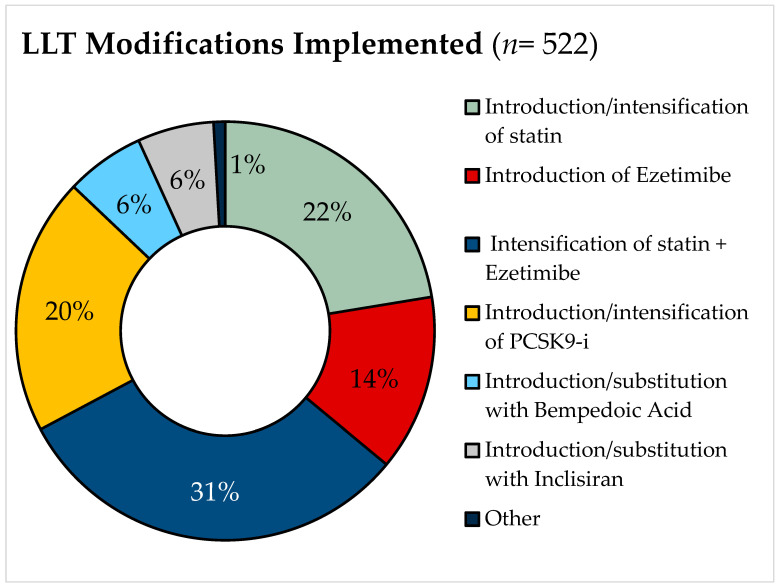
Types of lipid-lowering therapy (LLT) modifications and implementations prescribed. PCSK9-i: proprotein convertase subtilisin/kexin type 9 serine protease inhibitors.

Among the 522 patients not achieving the LDL-cholesterol target, changes in their LLTs were proposed, and various drug modifications/implementations were prescribed, as illustrated in Figure 3. According to the approach described in the Methods Section, only 221 patients (42.3%) were predicted to hypothetically reach the recommended LDL-cholesterol target following the proposed therapeutic changes.

## 4. Discussion

The main findings of this real-world study are as follows: (1) there is still a large proportion (58.7%) of patients with documented ASCVD not achieving the LDL-cholesterol goals recommended by the international guidelines; (2) combination therapy and newer LLTs (i.e., statin plus ezetimibe, the addition of bempedoic acid, PCSK9i or inclisiran) are still underused, despite current evidence of their higher efficacy compared to monotherapy with moderate to high-intensity statin; and (3) therapeutic inertia, i.e., no changes in LLT or changes that were unlikely to achieve the recommended LDL-cholesterol goal, was common among patients not achieving the LDL-cholesterol target.

Although intensive and early lipid-lowering in patients with ASCVD is safe and effective in clinical practice and associated with a reduction of residual CV risk [19,20,21,22], only 43.6% of patients in this multicenter study achieved the LDL target of ≤55 mg/dL, and a mere only 18.2% of those with a recommended target of ≤40 mg/dL (i.e., patients with multiple events within 2 years) reached these goals. These findings are in line with other European studies, such as the Da Vinci [2,3] and SANTORINI studies [4,23], which have similarly highlighted widespread challenges in achieving LDL-cholesterol targets across Europe. In the SANTORINI study [4], the proportion of patients achieving LDL-cholesterol levels below 55 mg/dL was approximately 20%, indicating a substantial number of high-risk patients still remain inadequately treated; a similar proportion, 18%, was reported from a recent systematic review [24]. However, the consistently higher percentage of patients achieving LDL-cholesterol targets in our study population, compared to the previously cited studies and registries, is encouraging and could be attributed to several factors, including a longer time distance from EAS/ESC guidelines publication, permitting the implementation of recommendations, and, therefore, a larger use of combination LLT versus monotherapy. The potential role of time elapsed since the EAS/ESC guidelines publication is further supported by our finding that patients evaluated clinically in the first half of 2024 achieved the LDL-cholesterol target more frequently compared to those evaluated in 2023 (44.6% vs. 39.4%, *p* < 0.03).

Therapeutic inertia in the management of dyslipidemia is a crucial challenge in secondary cardiovascular prevention [25] and emerged as a major contributor to the failure to achieve LDL-cholesterol targets in our cohort. Among the patients who did not reach their LDL-cholesterol goal, more than half (51.4%) did not have any changes made to their LLTs. This lack of treatment adjustment occurred despite the presence of multiple therapeutic options capable of significantly lowering LDL-cholesterol levels. For instance, combination therapy with statins, ezetimibe, and PCSK9 inhibitors has been shown to result in marked reductions in LDL cholesterol [26,27], yet many patients remained on monotherapy or low-intensity statins. The observed inertia was particularly prominent in older patients, those with a longer time since their initial ASCVD event, and patients with multiple events within a two-year period, who are recommended to achieve the more stringent LDL target of ≤40 mg/dL. The decision to not modify therapy in a significant number of patients likely reflects both clinician-related and patient-related factors. Clinicians may hesitate to escalate therapy due to concerns about side effects, polypharmacy, or perceived patient preferences. On the other hand, some patients may be reluctant to add new medications or intensify their current regimens due to concerns about costs, adherence, or potential side effects [7,28,29]. Additionally, healthcare access issues, particularly in regions with limited availability of newer LLTs (such as PCSK9 inhibitors), may further contribute to therapeutic inertia. Factors such as high costs, limited insurance coverage, and logistical barriers to accessing these therapies often make them inaccessible to many patients, even in cases where their clinical benefits and necessity are well-established [30,31].

Growing evidence suggests that combining a moderate-intensity statin with ezetimibe offers an effective and safe alternative to traditional high-intensity statin monotherapy [32,33,34]. This approach may also enhance therapeutic adherence by minimizing the side effects more commonly associated with high-intensity statins [35]. Similarly, hospital discharge following ASCVD events represents a critical juncture for ensuring long-term adherence to evidence-based therapies [36]. Evidence indicates that patients prescribed optimized medical therapy at discharge are significantly more likely to maintain their medication regimens, underscoring the need for stringent medication reconciliation and initiation of preventive therapies during this period [37].

Evaluating the appropriateness of LLT modifications is a complex task, as highlighted by the limited number of studies to date that have attempted to address this issue [38]. In our study, among the 522 patients for whom changes in LLTs were proposed, only 42.3% were deemed likely to achieve their LDL-cholesterol target following the recommended adjustments. This suggests that many of the proposed therapy modifications were insufficient or not appropriately tailored to the patient’s needs. For instance, simply doubling the dose of a statin for patients who are significantly above their LDL target is unlikely to produce the necessary LDL reduction. More aggressive approaches, such as the addition of ezetimibe, bempedoic acid PCSK9 inhibitors, or inclisiran, are often required but were underutilized in our study population. Indeed, an interesting simulation study revealed that achieving LDL cholesterol targets would require the introduction of PCSK9 inhibitors in up to 50% of secondary prevention patients, ultimately enabling over 91% of them to theoretically reach these goals [39]. Finally, it is possible that the older age of our study population plays a crucial role in underlying therapeutic inertia [40]. Navar AM et al. [41] reported that increasing age was associated with lower odds of statin use (odds ratio, 0.79 per 5-year increase at 60 years old [95% CI, 0.78–0.81]).

Our analysis suggests that a more systematic approach to adjusting LLTs could help overcome therapeutic inertia and improve the appropriateness of therapy changes. Specifically, we propose that routinely calculating the percentage distance from the LDL-cholesterol target could be a valuable supportive tool for guiding clinical decision-making. By expressing the patient’s LDL-cholesterol in relation to the guideline-recommended target, clinicians could more easily identify the need for therapy adjustments and determine the most appropriate modifications. For instance, a patient with an LDL-cholesterol level of 70 mg/dL and a target of 55 mg/dL is 21% away from their goal, indicating that a simple doubling of the statin dose (which provides only a 6% additional LDL reduction) would be insufficient. In such cases, the addition of ezetimibe or a PCSK9 inhibitor, which offers reductions of 20% and 50%, respectively, would be far more effective. The routine use of percentage distance from LDL-cholesterol target, ideally incorporated into electronic health records, could facilitate timely and appropriate adjustments to LLTs. This approach could reduce clinician hesitation and provide a clear rationale for intensifying therapy, thereby helping to close the gap between clinical guidelines and real-world practice. Moreover, such tools could empower patients by providing them with a clearer understanding of their LDL-cholesterol goals and the importance of maintaining optimal lipid levels to reduce their risk of future cardiovascular events [42,43].

### Limitations

The cross-sectional nature of the study does not allow us to document the real effects of changes in LLTs prescribed to patients initially not at the LDL-cholesterol target, as a subsequent lipid control measurement was not available. Accordingly, the effects of therapy intensification were estimated based on the efficacy of drugs as reported in the literature and on the levels of pre-treatment LDL-cholesterol, calculated using correction formulas. There is a large inter-individual variation in the response to LLTs, and predicted LDL-cholesterol often differs from measured values [44,45]. Consequently, the percentage of patients for whom therapy intensification would achieve the LDL-cholesterol target may have been under- or overestimated. Taking also into account that only data on pharmacological therapy modifications were available, while lifestyle interventions, which could potentially be sufficient in certain selected cases (e.g., when the distance from the LDL-cholesterol target is <6%), were not considered. Moreover, the cross-sectional nature of the study, with a single measurement of LDL-cholesterol at varying intervals from the first ASCVD event, does not rule out that patients may have reached the target at some point with the prescribed therapy, followed by fluctuations in LDL-cholesterol variability due to adherence, dietary, or behavioral changes [46]. Additionally, this study does not permit analysis of factors related to therapeutic inertia and adherence in individual patients. Finally, neither lipoprotein(a) nor inflammation data (e.g., interleukins) were available to further assess residual risk.

## 5. Conclusions

In conclusion, our study highlights the significant gap in achieving LDL-cholesterol targets in patients at very high cardiovascular risk and the critical role that therapeutic inertia plays in this shortfall. By adopting more structured and systematic approaches to therapy adjustments, such as calculating the percentage distance from LDL-cholesterol targets, clinicians may be better equipped to make informed, timely decisions that can improve patient outcomes. However, it is also essential to ensure that the proposed therapy modifications are appropriate and sufficiently aggressive to achieve the desired LDL-cholesterol reductions. Future efforts should focus on integrating these strategies into routine clinical practice, potentially supported by technological tools to streamline decision-making and optimize lipid management.

## Figures and Tables

**Table 1:** Demographic and clinical characteristics of included patients. SD: standard deviation, CCS: chronic coronary syndrome, ACS: acute coronary syndrome, TIA: transient ischemic attack, PAD: peripheral atherosclerotic disease.

Demographic and Clinical Characteristics	
Number of patients	1909
Age ± SD	68 ± 11.4
- More than 75 years old (%)	- 517 (27.1)
Female gender (%)	402 (21.1)
Clinical event (%)	1909 (100)
- CCS	- 392 (20.5)
- Post-ACS	- 1451 (76.0)
- Stroke/TIA	- 99 (5.2)
- PAD	- 268 (14.0)
- Combination (at least 2)	- 285 (14.9)
Multiple events < 2 years (%)	170 (8.9)
Multivessel coronary artery disease (%)	1018 (55.2)
Time since first event (months ± SD)	77 ± 87
Event < 1 year (%)	485 (26.6)
Hypertension (%)	1414 (74.1)
Diabetes (%)	506 (26.6)
Dyslipidaemia (%)	1604 (84.0)
Chronic renal disease (%)	329 (17.2)
Obesity (%), data from *n* = 968	184 (19.0)
Smoking status (%)	
- Never	- 920 (48.2)
- Former	- 732 (38.3)
- Active	- 257 (13.5)
Symptoms (%), data from *n* = 1604	239 (14.9)
- Angina	- 54 (3.4)
- Dyspnea	- 190 (12.0)
- Claudication	- 31 (2.0)
Atrial fibrillation (%)	237 (12.4)
Cardiac rehabilitation (%), data from *n* = 1209	589 (48.7%)

**Table 2:** Lipid data and lipid-lowering therapies of included patients. SD: standard deviation, LDL-c: low-density lipoprotein cholesterol, HDL-c: high-density lipoprotein, PCSK9-i: proprotein convertase subtilisin/kexin type 9 serine protease inhibitors, PUFA: Polyunsaturated Fatty Acids.

Lipid Data	
Number of patients	1909
Total cholesterol (mg/dL) ± SD, *n* = 1487	137.6 ± 41
LDL-c (mg/dL) ± SD	66.5 ± 33.6
HDL-c (mg/dL) ± SD, *n* = 1417	47.9 ± 12.8
Non HDL-c (mg/dL) ± SD, *n* = 1412	89.5 ± 38.7
Triglycerides (mg/dL) ± SD, *n* = 1439	118.6 ± 57.3
Patients with LDL-c target < 55 mg/dL (%):	1739 (91.1)
- Achieving Target	758 (43.6)
Patients with LDL-c target < 40 mg/dL (%):	170 (8.9)
- Achieving Target	31 (18.2)
Lipid-Lowering Data	
No therapy (%)	59 (3.1)
Statin only (%)	510 (26.7)
Ezetimibe only (%)	24 (1.3)
PCSK9-i only (%)	26 (1.3)
Bempedoic acid only (%)	0 (0)
Inclisiran only (%)	2 (0.1)
Statin + ezetimibe (%):	1046 (54.8)
- Single pill	- 826 (79.0)
Bempedoic acid + ezetimibe (%)	8 (0.4)
- Single pill	- 6 (75.0)
PCSK9-i + ezetimibe (%)	39 (2.0)
Statin + ezetimibe + PCSK9-i (%)	93 (4.9)
Bempedoic acid + PCSK9-i (%)	2 (0.1)
Fibrates (%)	20 (1.0)
PUFA (%)	125 (6.5)

**Table 3:** Comparison between patients who achieved versus not achieved LDL-c Target. SD: standard deviation, LDL-c: low-density lipoprotein cholesterol, HDL-c: high-density lipoprotein, PCSK9-i: proprotein convertase subtilisin/kexin type 9 serine protease inhibitors, PUFA: Polyunsaturated Fatty Acids. Bold = *p* value < 0.05.

	Achieving LDL-C Target	Not Achieving LDL-C Target	*p* Value
Number of patients (%)	789 (41.3)	1120 (58.7)	
Age ± SD	66.9 ± 10.9	68.9 ± 11.7	**<0.001**
Female gender (%)	107 (13.6)	295 (26.3)	**<0.001**
Multiple events < 2 years (%)	31 (3.9)	139 (12.4)	**<0.001**
Multivessel disease (%)	447 (57.3)	571 (53.7)	**0.014**
Time since event (months) ± SD	70 ± 84	72 ± 86	**0.005**
Event < 1 year (%)	234 (31.0)	251 (23.4)	**<0.001**
Hypertension (%)	577 (73.1)	837 (74.7)	0.432
Diabetes (%)	244 (30.9)	262 (23.4)	**<0.001**
Chronic renal disease (%)	122 (15.5)	207 (18.5)	0.085
Obesity (%),(*n* = 968)	68 (19.3)	116 (28.2)	**0.004**
Symptoms (%), *n* = 1604	92 (14.3)	147 (15.3)	0.180
Cardiac rehabilitation (%), *n* = 1209	317 (66.3)	272 (37.2)	**<0.001**
Total cholesterol (mg/dL) ± SD, *n* = 1487	108.2 ± 19	158.9 ± 39	**<0.001**
LDL-c (mg/dL) ± SD	40.8 ± 10.8	84.6 ± 32.3	**<0.001**
Absolute distance from LDL-C target (mg/dL) ± SD	−13.6 ±10.5	31.5 ± 31.9	**<0.001**
Percentage distance from LDL-C target (%) ± SD	−53.2 ± 98.9	31.5 ± 18.5	**<0.001**
HDL-c (mg/dL) ± SD, *n* = 1417	45.8 ± 12.1	49.4 ± 13.1	**<0.001**
Not HDL-c (mg/dL) ± SD, *n* = 1412	62.2 ± 16.6	110.2 ± 37.9	**<0.001**
Triglycerides (mg/dL) ± SD, *n* = 1439	114.1 ± 58.1	122.0 ± 56.4	**0.009**
No therapy (%)	5 (0.6)	55 (4.9)	**<0.001**
Statin only (%)	116 (14.7)	394 (35.2)	**<0.001**
Ezetimibe only (%)	1 (0.1)	23 (2.1)	**0.001**
PCSK9-i only (%)	10 (1.3)	16 (1.4)	0.853
Statin + ezetimibe (%):	526 (66.7)	520 (46.4)	**<0.001**
- Single pill	- 434 (82.5)	- 392 (75.4)	- **0.005**
PCSK9-i + ezetimibe (%)	17 (2.1)	22 (2.0)	0.879
Statin + ezetimibe + PCSK9-i (%)	74 (9.4)	19 (1.7)	**<0.001**
PUFA (%)	40 (5.1)	85 (7.6)	**0.03**

**Table 4:** Results of the univariate and multivariate analyses for identifying predictors of achieving LDL-cholesterol targets. LDL-c: low-density lipoprotein cholesterol, ASCVD: atherosclerotic cardiovascular disease, PCSK9-i: proprotein convertase subtilisin/kexin type 9 serine protease inhibitors, β: coefficient, CI: confidence interval. Bold = *p* value < 0.05.

Predictors of Achieving LDL-c Targets	Univariable		Multivariable	
	β [95% CI]	*p* Value	β [95% CI]	*p* Value
Male gender	6.82 [5.20, 8.44]	**<0.001**	3.78 [2.64, 4.92]	**<0.001**
Acute coronary syndrome	4.85 [3.60, 6.10]	**<0.001**	1.96 [0.00, 3.92]	**0.05**
ASCVD event < 1 year	3.61 [2.20, 5.02]	**<0.001**	−1.41 [−3.34, 0.52]	0.158
Multiple events < 2 years	−6.47 [−7.90, −5.04]	**<0.001**	−4.49 [−5.85, −3.13]	**<0.001**
Cardiac rehabilitation	10.3 [8.50, 12.1]	**<0.001**	3.76 [2.62, 4.90]	**<0.001**
Diabetes	3.72 [2.10, 5.34]	**<0.001**	2.38 [0.40, 4.36]	**0.018**
Dyslipidaemia	−3.55 [−4.90, −2.20]	**<0.001**	−3.57 [−5.04, −2.10]	**<0.001**
Obesity	−2.88 [−4.82, −0.94]	**0.004**	−1.37 [−3.33, 0.59]	0.170
No therapy	−5.24 [−6.80, −3.68]	**<0.001**	−2.80 [−4.72, −0.88]	**0.005**
Statin only	−10.8 [−12.5, −9.10]	**<0.001**	−5.77 [−7.21, −4.33]	**<0.001**
Ezetimibe only	−4.19 [−5.62, −2.76]	**<0.001**	−2.82 [−4.32, −1.32]	**<0.001**
Statin + ezetimibe	8.51 [6.74, 10.3]	**<0.001**	−0.90 [−2.86, 1.06]	0.371
Statin + ezetimibe + PCSK9-i	7.80 [6.10, 9.50]	**<0.001**	2.37 [0.42, 4.32]	**0.018**

**Table 5:** Comparison between patients who underwent versus those who did not undergo lipid-lowering therapy modifications. SD: standard deviation, LDL-c: low-density lipoprotein cholesterol, HDL-c: high-density lipoprotein, PCSK9-i: proprotein convertase subtilisin/kexin type 9 serine protease inhibitors, PUFA: polyunsaturated fatty acids. Bold = *p* value < 0.05.

Not Achieving LDL-C Target Patients (*n* = 1074)	Drug Modification	No Drug Modification	*p* Value
Number of patients (%)	522 (48.6)	552 (51.4)	
Age ± SD	68.0 ± 10.9	70.3 ± 11.0	**<0.001**
Female gender (%)	149 (28.5)	132 (23.9)	0.084
Multiple events < 2 years (%)	35 (6.7)	93 (16.8)	**<0.001**
Multivessel disease (%)	246 (47.1)	302 (54.7)	**0.013**
Time since event (months) ± SD	76 ± 85	89 ± 64	**0.013**
Event < 1 year (%)	150 (29.5)	84 (15.5)	**<0.001**
Cardiac rehabilitation (%), *n* = 1209	148 (39.6)	84 (26.5)	**<0.001**
Total cholesterol (mg/dL) ± SD, *n* = 1487	171.8 ± 43	145.4 ± 28	**<0.001**
LDL-c (mg/dL) ± SD	96.3 ± 37.3	74.6 ± 23.2	**<0.001**
Absolute distance from LDL-C target (mg/dL) ± SD	42.3 ±37.4	22.1 ± 22.9	**<0.001**
Percentage distance from LDL-C target (%) ± SD	38.1 ± 17.8	25.1 ± 17.0	**<0.001**
HDL-c (mg/dL) ± SD, *n* = 1417	49.5 ± 11.3	49.3 ± 14.8	0.814
Not HDL-c (mg/dL) ± SD, *n* = 1412	123.1 ± 42.8	96.4 ± 25.4	**<0.001**
Triglycerides (mg/dL) ± SD, *n* = 1439	126.5 ± 58.6	117.0 ± 53.4	**0.016**
No therapy (%)	33 (6.3)	16 (2.3)	**0.001**
Statin only (%)	223 (42.7)	161(29.2)	**<0.001**
Ezetimibe only (%)	19 (3.6)	4 (0.2)	**<0.001**
PCSK9-i only (%)	8 (1.5)	8 (1.4)	0.891
Statin + ezetimibe (%):	186 (35.2)	306 (55.4)	**<0.001**
- Single pill	- 143(76.9)	- 248 (81.0)	- 0.276
PCSK9-i + ezetimibe (%)	8 (1.5)	14 (2.5)	0.244
Statin + ezetimibe + PCSK9-i (%)	4 (0.8)	14 (2.5)	**0.03**
PUFA (%)	36 (6.9)	48 (8.7)	0.273

**Table 6:** Results of the univariate and multivariate analyses for identifying predictors of lipid-lowering therapy (LLT) modifications. LDL-c: low-density lipoprotein cholesterol, ASCVD: atherosclerotic cardiovascular disease, PCSK9-i: proprotein convertase subtilisin/kexin type 9 serine protease inhibitors, β: coefficient, CI: confidence interval. Bold = *p* value < 0.05.

Predictors of Modifying LLT	Univariable		Multivariable	
	β [95% CI]	*p* Value	β [95% CI]	*p* Value
Age	−3.36 [−4.50, −2.22]	**<0.001**	−2.56 [−3.80, −1.32]	**0.011**
Multivessel disease	−2.49 [−3.85, −1.13]	**0.013**	0.89 [−0.55, 2.33]	0.370
ASCVD event < 1 year	5.51 [4.20, 6.82]	**<0.001**	3.91 [2.85, 4.97]	**<0.001**
Multiple events < 2 years	−5.19 [-6.70, −3.68]	**<0.001**	−4.74 [−6.20, −3.28]	**<0.001**
Time since event	−2.48 [−3.75, −1.21]	**0.013**	0.51 [−0.85, 1.87]	0.610
Cardiac rehabilitation	3.66 [2.75, 4.57]	**<0.001**	5.35 [3.97, 6.73]	**<0.001**
Dyslipidaemia	2.33 [0.36, 4.30]	**0.020**	−1.21 [−3.15, 0.73]	0.229
Chronic renal sisease	−2.83 [−4.30, −1.36]	**0.005**	−0.75 [−2.10, 0.60]	0.452
LDL-c	11.5 [8.75, 14.25]	**<0.001**	1.99 [−0.75, 4.73]	0.146
Percentage distance from LDL-C target	12.26 [9.80, 14.72]	**<0.001**	7.53 [5.25, 9.81]	**<0.001**
No therapy	2.69 [0.75, 4.63]	**0.007**	1.38 [−0.85, 3.61]	0.169
Statin only	5.12 [3.50, 6.74]	**<0.001**	1.18 [−0.75, 3.11]	0.239
Ezetimibe only	3.62 [1.10, 6.14]	**<0.001**	0.29 [−1.45, 2.03]	0.771
Statin + ezetimibe	−6.64 [−8.50, −4.78]	**<0.001**	−0.68 [−2.30, 0.94]	0.497
Statin + ezetimibe + PCSK9-i	−2.26 [−3.75, −0.77]	**0.024**	−1.05 [−2.85, 0.75]	0.296

## Data Availability

The raw data supporting the conclusions of this article will be made available by the authors on request.
